# Mortality rate and predictors of time to death in children with severe acute malnutrition treated in Felege-Hiwot Referral Hospital Bahir Dar, Northwest Ethiopia

**DOI:** 10.1186/s13104-019-4467-x

**Published:** 2019-07-15

**Authors:** Hanna Demelash Desyibelew, Adhanom Gebreegziabher Baraki, Abel Fekadu Dadi

**Affiliations:** 1 Department of Public Health Nutrition, College of Medicine and Health Sciences, Bahirdar University, Bahir Dar, Ethiopia; 2 Department of Epidemiology and Biostatistics, College of Medicine and Health Sciences, University of Gondar, Gondar, Ethiopia; 3 Flinders University, Health Sciences Building, Sturt Road, Bedford Park, Adelaide, SA 5001 Australia

**Keywords:** Sever acute malnutrition, Mortality, Time to death

## Abstract

**Objectives:**

This study aimed to determine mortality rate, time to death and factors affecting the time to death among children with severe acute malnutrition admitted to therapeutic feeding unit of Felege Hiwot Referral Hospital, Bahirdar.

**Result:**

A total of 401 children with severe acute malnutrition who were admitted to therapeutic feeding units from September 2012 to January 2016 were included in the study. The incidence of death rate was 8.47% (95% CI 6.11%, 11.65%). The median time to death was 3 days (Inter Quartile Range of 4 days). Children’s of age > 24 months (AHR = 0.27; 95% CI 0.1, 0.73), fully vaccinated status (AHR = 0.16; 95% CI 0.07, 0.36), HIV infection (AHR = 3.82; 95% CI 1.3, 11.15) and congestive heart failure (AHR = 6.98; 95% CI 2.42, 20.09) were significant predictors of mortality among children admitted for severe acute malnutrition.

## Introduction

In Ethiopia, SAM is the third leading cause of mortality [1–3] in under-five children and more than one-fourth of deaths are occurring during hospital admission [1, 4]. The mortality rate due to SAM in Ethiopia ranges from 3.8 to 12.5% [1–4]. The last four Ethiopian Demographic Health Survey (EDHS) shows an increasing trend in the proportion of children who are severely wasted: 1% in EDHS 2000 [5], 2% in EDHS 2005 [6], 3% in EDHS 2011 [7] and 3% in EDHS 2016 [8]. However, in Amhara region, a steady reduction in severe wasting was observed though it is not adequate [6–8].

Severe acute malnutrition in early life can continue to manifest in the later life and can results in disability, diet related non-communicable diseases, and high economic burden [9–12]. Inversely, improving child nutrition can have a powerful effect across multiple aspects of development, environmental sustainability, and peace and stability [10, 13, 14]. The continuing burden of high mortality rate was determined by a faulty case management, co-morbidities (TB, HIV), severity of illness at presentation for treatment and geographic and socio-economic changes [1–4, 15].

High mortality rate in therapeutic feeding centers is the main challenge of successful treatment outcome in developing countries like Ethiopia and generating a local based data for reason to death is highly important to meet the goal of therapeutic feeding centers. This study tried to identify key characteristics of SAM children with poor outcomes. This study have also estimated the mortality rate, and identified predictors of death among children admitted in Felege Hiwot Referral Hospital therapeutic feeding unit.

## Main text

### Methods

Retrospective record review of under-five children’s admitted at Felege Hiwot Specialized Hospital therapeutic feeding center was done. We included all under-five children with severe acute malnutrition who were admitted and treated at the inpatient therapeutic feeding unit from September 2012 to January 2016. The Hospital serves for more than 5 million people since 1962. Children’s with age 6 to 59 months who presented with a nutritional bilateral pitting edema and/or weight for height Z-scores (WHZ) < − 3 standard deviations (SD) were involved in the chart review.

The minimum sample size required using an exponential model considering the following assumptions; type I error 5%, power of 90% and Children older than 3 years [AHR = 0.67] [16] was 366. After adding a 10% contingency for missing and incomplete data, we have got 403. However, we found a record for only 401 children’s between September 2012 to January 2016 and we included all of them in the study. We used the following STATA 14 command for determining the sample size.

$$ \begin{aligned} &Power\;exponential,\;hratio\;\left( {0.69} \right)\;effect\;\left( {hratio} \right)\;\\ &\quad power\;\left( {0.9} \right)\;nratio(2) \end{aligned} $$

A well designed and pre-tested data extraction form was used to extract a required information from a patient card. A 2 day practical training was given for data collectors on the objective of the study, how to review registration log books and patient’s chart, and confidentiality of the data. Health professionals working with a pediatric ward were involved in extracting the data under a principal investigators close supervision.

The collected data were checked for inconsistencies, coding error, completeness, accuracy, clarity and missing values. Summary measures such as counts, percentages, medians, Inter-Quartile ranges, means, and standard deviations were generated. Time to death was defined as the time from the start of treatment to death while he/she was in the program. Censored was declared when the children was not died until the end of our follow up or cured or drop out of treatment or transferred to another treatment center.

Log rank test was applied to compare survival time. The parametric survival analysis using Gompertz regression was used to identify predictors of time to death. Crude and adjusted hazard ratios with their 95% confidence interval (CI) were estimated and P-value less than 0.05 were used to declare the presence of significant association. All statistical analysis was done using Stata 14.0.

### Result

#### Socio-demographics, co-morbidity treatment related characteristics

A total of 401 children with severe acute malnutrition were admitted to therapeutic feeding units from September 2012 to January 2016. Out of these 401 reviewed records, 223 (55.6%) were male. Majority 255 (63.6%) of the children were between the age of 6 to 24 months. The median age was 20 months (IQR = 20). Two hundred fifty-three (63.1%) of the children had sever wasting and the other 148 (36.9%) had edema. Majority of wasted (92.9%) and edematous (86.5%) SAM children had complications at admission. Diarrhea (36.2%) and pneumonia (39.2%) were the most prevalent co-morbidities (Table 1).

**Table 1:** Baseline socio-demographics and co-morbidity characteristics of SAM children admitted at Felege Hiwot Referal Hospital therapeutic feeding center, Northwest, Ethiopia, 2016 (n = 401)

Variables	Category	Death frequency (%)	Censored frequency (%)
Sex	Male	26 (11.66)	197 (88.34)
	Female	8 (4.49)	170 (95.51)
Age	6–24	26 (10.20)	229 (89.80)
	25–59	8 (5.48)	138 (94.52)
Residence	Urban	9 (7.2)	116 (92.8)
	Rural	25 (9.06)	25 1 (90.94)
Breast feeding	Yes	31 (8.18)	348 (91.82)
	No	3 (13.64)	19 (86.36)
Vaccination for age	Unvaccinated/partial	21 (28.38)	53 (71.62)
	Fully vaccinated	13 (3.98)	314 (96.02)
Diarrhea	Yes	13 (8.97)	132 (91.03)
	No	21 (8.20)	235 (91.80)
Tuberculosis	Yes	6 (16.22)	31 (83.78)
	No	28 (7.69)	336 (92.31)
HIV	Yes	7 (26.92)	19 (73.08)
	No	27 (7.20)	348 (92.8)
Pneumonia	Yes	18 (11.46)	139 (88.54)
	No	16 (6.56)	228 (93.46)
URTI	Yes	5 (14.72)	29 (85.28)
	No	29 (7.90)	338 (92.10)
Measles	Yes	7 (17.07)	34 (82.93)
	No	23 (6.59)	326 (93.41)
CHF	Yes	6 (20.69)	23 (79.31)
	No	28 (7.53)	344 (92.47)

Most of the children have received routine medications. The most commonly administered routine medications were ampicillin and gentamycin 303 (75.6%), vitamin-A 285 (71%) and folic acid 381 (95%). Large proportion, 116 (80%) of children who presented with diarrhea have received Zinc in addition to routine medications. Across the treatment time different therapeutic foods were given. F75 was given for 393 (98%); F100 dilute was given for 315 (78.6%) and F100 was given for 273 (68%) of the children. All the children were fed 6 times per day.

#### Mortality rate and predictors of time to death

**Fig. 1:**
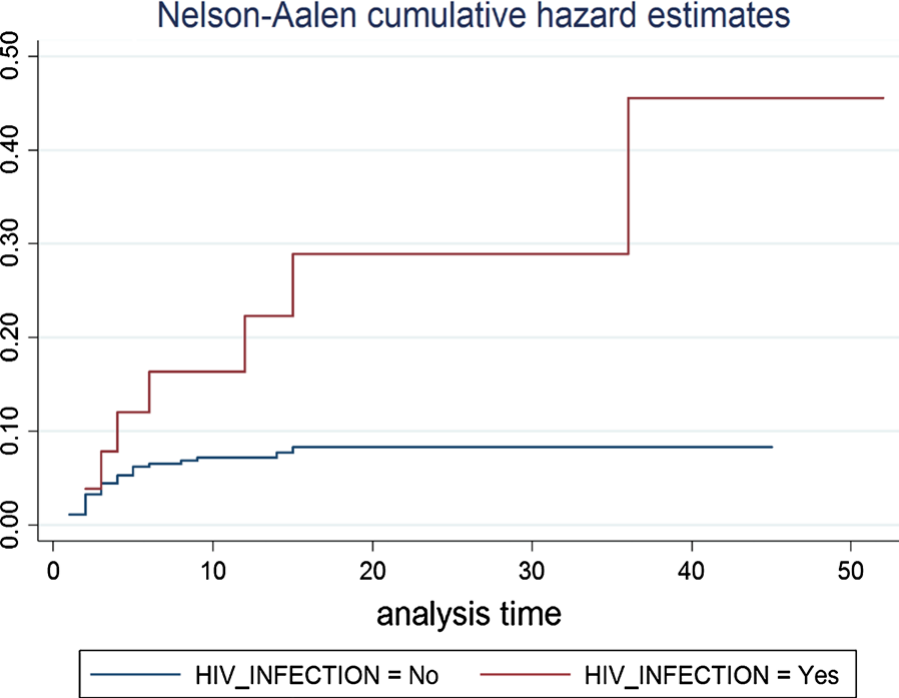
Nelson–Aalen cumulative hazard estimate for HIV infected and HIV uninfected SAM children in Felege Hiwot Referral Hospital, 2016

We estimated a mortality rate of 8.47% (95% CI: 6.11, 11.65). The median time to death was 3 days (IQR 4 days). The cumulative hazard estimate of death was found to be higher among HIV infected children when compared to their counter parts (Fig. 1).

The overall Schoenfeld global test of the full model was checked for Proportional Hazard (PH) assumption and it was met (P-value = 0.5034). Different models with different distributional assumptions were also compared to identify the best model and finally a model with the lowest AIC, which is the parametric survival test with Gompertz distribution was used to generate the estimates. In bi-variable parametric survival Gompertz regression; sex, age, vaccination status, tuberculosis, HIV/AIDS, pneumonia, URTI, measles and CHF were found to be a significant predictors of time to death at a P value < 0.2. However, in the final multivariable model; age, vaccination status, HIV/AIDS, and CHF were significantly associated with time to death.

The hazard of death among children above 2 years old was 73% (AHR = 0.27; 95% CI 0.1, 0.73) reduced as compared to those under 2 years. Fully vaccinated children had 84% (AHR = 0.16; 95% CI 0.07, 0.36) lower hazard of death as compared to unvaccinated/partially vaccinated children. HIV infected children had 3.82 (AHR = 3.82; 95% CI 1.3, 11.15) times higher hazard of death as compared to their counter parts. Children who had congestive heart failure had 6.98 (AHR = 6.98; 95% CI 2.42, 20.09) times higher hazard of death as compared to those children without congestive heart failure (Table 2).

**Table 2:** The bi-variable and multivariable parametric gompertz regression analysis of time to death among children with SAM at Felege Hiwot Specialized Hospital Northwest, Ethiopia

Variables	Died	Censored	CHR (95% CI)	AHR (95% CI)
	Number (%)	Number (%)		
Sex				
Male	26 (11.66)	197 (88.34)	1	1
Female	8 (4.49)	170 (95.51)	0.37 (0.17, 0.81)	0.39 (0.16, 1.00)
Age group				
6–24	26 (10.20)	229 (89.80)	1	1
25–59	8 (5.48)	138 (94.52)	0.47 (0.21, 1.04)	0.27 (0.10, 0.73)*
Vaccination status				
Unvaccinated/partial	21 (28.38)	53 (71.62)	1	1
Fully vaccinated	13 (3.98)	314 (96.02)	0.12 (0.06, 0.24)	0.16 (0.07, 0.36)*
Tuberculosis				
No	28 (7.69)	336 (92.31)	1	1
Yes	6 (16.22)	31 (83.78)	1.94 (0.80, 4.69)	0.91 (0.27, 3.11)
HIV				
No	27 (7.20)	348 (92.8)	1	1
Yes	7 (26.92)	19 (73.08)	3.54 (1.54, 8.14)	3.82 (1.3, 11.15)*
Pneumonia				
No	16 (6.56)	228 (93.46)	1	1
Yes	18 (11.46)	139 (88.54)	1.73 (0.88, 3.39)	2.00 (0.88, 4.56)
URTI				
No	29 (7.90)	338 (92.10)	1	1
Yes	5 (14.72)	29 (85.28)	2.11 (0.82, 5.47)	2.42 (0.76, 7.72)
Measles				
No	23 (6.59)	326 (93.41)	1	1
Yes	7 (17.07)	34 (82.93)	2.43 (1.04, 5.66)	0.73 (0.27, 1.99)
CHF				
No	28 (7.53)	344 (92.47)	1	1
Yes	6 (20.69)	23 (79.31)	3.51 (1.45, 8.47)	6.98 (2.42, 20.09)*

* P value < 0.05

### Discussion

Thirty-four under-five children (8.47%) were died during the follow up period. The median time to death after admission was 3 days. Factors like age, vaccination status, HIV/AIDS, and CHF were significantly associated with time to death in children with severe acute malnutrition admitted for therapeutic feeding unit.

A death rate that we have estimated is expected according to the minimum international standard of acute malnutrition management [8] and it is lower than a study conducted in Uganda [6] and Zambia [11] but it is higher than a study conducted in southern Ethiopia [17]. The possible reason for this discrepancy could be variation in compliance to the standard treatment guideline or and severity of malnutrition at the time of their admission.

The median time to death was 3 days after admission. This finding is faster when compared with a study conducted in Jimma university specialized hospital [18]. But our finding is consistent with other several studies that reported a high death rate at early period of admission [6, 19, 20]. This might be correlated with late presentation of the children to the therapeutic feeding center or presentation with severe condition or difference in patient profile or it might be related with an in efficient case management at the health facilities, which all of this patient and health services related characteristics might affect children’s response to the treatment.

Children under the age of 24 months had higher hazard of death as compared to their counter parts. This finding was in agreement with other studies [5, 18, 21], the possible reason could be these children were more likely to have a less developed immunity, less likely to adequately respond for the treatment, more likely to develop infection and to have inadequate nutrition. Unvaccinated/partially vaccinated children had higher hazard of death as compared to those fully vaccinated. This could be due to the occurrence of more vaccine preventable co-morbid situations like measles and diarrheal disease that facilitate the risk of death among children who were unvaccinated than fully vaccinated [22].

We found HIV co-infected children to have higher hazard of death as compared to their counterparts. This finding is consistent with other studies [6, 18, 21, 23], this could be due to the fact that children’s with HIV infection are more likely to have chronic diarrhea that impairs nutrient absorption or it could be due to other opportunistic oral lesions that makes feeding difficult [24].

The hazard of death among children with CHF was higher when compared with children with no CHF, which also published in other similar study [18]. Weak pulse volume that is common in CHF patients [19] and pulmonary congestion that could be worsened by a high energy feeding [25] might increase their risk and time of death.

According to the findings of this study the mortality rate is < 10% which is appreciable for meeting the minimum international standard of acute malnutrition management, but further efforts also shall be done to reduce risk of death even more. Therefore especial attention shall be given for children who are under 2 years of age, HIV infected and those with heart failure. Full vaccination also must be enhanced to reduce death alongside with the mentioned risk reduction.

### Conclusion

The death rate in the study area is within the acceptable range but most of deaths occur early after admission. We found that children in younger, those unvaccinated, and those who had HIV infection and congestive heart failure were died faster as compared to their counterparts. So emphasis must be given for newly admitted children and those with the identified risk factors.

## Limitations

The possible limitation of this study is that since it is a record review, it failed to consider a broad range of factors like socio-demographic characteristics, biochemical and patient management related factors that we might introduced a high array of missing confounders. As such, the interpretation and application of the finding for decision and policy direction should account for these inherent limitations of the study.

## Data Availability

The data upon which the result based could be accessed by a reasonable request made to the corresponding author.

## Abbreviations

AIDS: acquired immunodeficiency syndrome
AHR: adjusted hazard ratio
AIC: Akaike’s information criteria
CHF: congestive heart failure
CHR: crude hazard ratio
CI: confidence interval
EDHS: Ethiopian Demographic and Health Survey
HIV: human immunodeficiency virus
IQR: inter-quartile range
MUAC: mid upper arm circumference
PH: proportional hazard
SAM: sever acute malnutrition
SD: standard deviation
TB: tuberculosis
URTI: upper respiratory tract infection
WHO: World Health Organization
WHZ: weight for height Z score
